# Worldwide Occurrence of Beijing/W Strains of *Mycobacterium tuberculosis*: A Systematic Review

**DOI:** 10.3201/eid0808.020002

**Published:** 2002-08

**Authors:** Judith R. Glynn, Jennifer Whiteley, Pablo J. Bifani, Kristin Kremer, Dick van Soolingen

**Affiliations:** * London School of Hygiene and Tropical Medicine, London, UK; † Institut Pasteur de Lille, France; and ‡National Institute of Public Health and the Environment (RIVM), Bilthoven, the Netherlands

**Keywords:** *Mycobacterium tuberculosis*, Beijing family, molecular epidemiology, resistance, spread

## Abstract

Strains of the Beijing/W genotype family of *Mycobacterium tuberculosis* have caused large outbreaks of tuberculosis, sometimes involving multidrug resistance. This genetically highly conserved family of *M. tuberculosis* strains predominates in some geographic areas. We have conducted a systematic review of the published reports on these strains to determine their worldwide distribution, spread, and association with drug resistance. Sixteen studies reported prevalence of Beijing strains defined by spoligotyping; another 10 used other definitions. Beijing strains were most prevalent in Asia but were found worldwide. Associations with drug resistance varied: in New York, Cuba, Estonia, and Vietnam, Beijing strains were strongly associated with drug resistance, but elsewhere the association was weak or absent. Although few reports have measured trends in prevalence, the ubiquity of the Beijing strains and their frequent association with outbreaks and drug resistance underline their importance.

In the early 1990s, a multidrug-resistant *Mycobacterium tuberculosis* strain was identified in New York (1). This strain, designated “W,” which was associated with large institutional outbreaks of tuberculosis (TB) and many deaths, was later identified in other parts of the United States (2,3). In 1995, a large proportion of the *M. tuberculosis* strains in the Beijing area of China was reported to have mutually highly similar multi-banded IS*6110* restriction fragment-length polymorphism (RFLP) patterns; these “Beijing” strains were also present in many other populations (4).

The New York City multidrug-resistant “W” strain was, in the second half of the 1990s, recognized as a member of the “Beijing” genotype family of *M. tuberculosis* strains (5–7). The W strain is recognized by a specific IS*6110* fingerprint pattern, by multiplex polymerase chain reaction (PCR) targeted at specific insertions, or both (2,3). W family strains have IS*6110* patterns closely related to that of W, although the degree of similarity in different studies has not always been specified. Beijing strains, including the W variants, have an insertion of IS*6110* in the genomic dnaA-dnaN locus (5,7). All W family strains have a characteristic spoligotype that is shared with the whole Beijing family of strains and seems to be specific for this family (4,8,9). Spoligotyping is based on DNA polymorphism in the direct repeat region, and “Beijing” spoligotypes only contain spacers 35–43.

The combination of a widespread family of strains and, in some situations, the association with multidrug resistance has led to concern that these strains may be spreading and may have a predilection for acquiring drug resistance. Many recent studies have recorded “Beijing-like” or “W-like” strains. We have conducted a systematic review of published reports to assess how widespread the family of strains is, whether there is any evidence that it is spreading, and whether it is associated with drug resistance.

## Methods

Relevant studies were identified through computerized searches of Medline (January 1, 1990–November 1, 2001) and PubMed (January 1, 2000–November 1, 2001), manually searching key journals, searching the Internet, and cross-checking references with collections of articles on Beijing strains compiled by researchers in the field. The computerized searches used both thesaurus and free-text terms to search for tuberculosis and any of the following: molecular epidemiology, DNA fingerprinting, DNA fingerprint*, typing, type, types, restriction fragment-length polymorphism, RFLP, spoligotyping, spoligotyp*, strain, and strains. The International Journal of Tuberculosis and Lung Disease, its predecessor Tuberculosis and Lung Disease, and the Journal of Clinical Microbiology were searched manually back to January 1990. A request for relevant articles was sent to all 32 participants in the European Union Concerted Action project on New Generation Genetic Markers and Techniques for the Epidemiology and Control of Tuberculosis. An Internet search, using Google, used the term “Beijing strain tuberculosis.” The reference lists of all included articles were searched for additional relevant studies.

Articles were included if they contained information allowing estimation of the proportion of TB patients included with the Beijing or W strains. Articles were excluded if they were limited to a particular outbreak, if they included only drug-resistant strains, or if <30 TB patients were included. Identified articles were subdivided into those that used spoligotyping to identify Beijing family strains and those that used other methods. Where spoligotypes were shown, estimates based on the spoligotype were used rather than any estimate given in the papers, using the proportion with spacers 35–43. Studies identifying only W strains or other W-like strains with a single IS*6110* fingerprint pattern will underestimate the prevalence of Beijing strains, since they identify only part of the family of strains. The method of patient selection was recorded when stated. In all studies, any evidence of changes over time or by age group or of any association between strain type and drug resistance was recorded.

## Results

Five thousand nineteen articles were selected from the initial search of Medline and PubMed. The titles and abstracts of these articles were scanned for relevant information, and 4,909 articles were rejected, leaving 110 articles for full text review. No further articles were identified by manual searching, but one recently published article was identified in the article collections that had not yet been indexed in the databases (10). One additional article was identified from reference list check that was published in a Vietnamese journal not indexed by Medline, EMBASE, or Web of Science, and we have been unable to locate it. Another article was found from an Internet search, in an electronic journal (11). Of the 112 articles reviewed in full, 26 fulfilled the inclusion criteria of this review, including 16 that gave results based on spoligotyping and several that reported results from more than one area (Tables 1,2; Figure). Studies that described patients who were apparently included in other reports have been excluded (31,32).

**Table 1:** Prevalence of Beijing family strains in studies that have used spoligotyping^a^

Reference	Setting	Yrs	Population	New TB or new + old	Prevalence Beijing strain N/N (%)
Asia					
12	Beijing and Hebei province, China	1956–1960	Stored lung biopsy samples from pneumonectomies	? Both	9/10 (90)
		1969–1970			8/9 (89)
		1979–1980			18/18 (100)
		1989–1990			10/12 (83)
		1956–1990			45/49 (92)
4	Beijing, China	1992–1994	? selection method	? Both	45/49 (92)
13	Hong Kong	1998–1999	Random sample	? New	337/500 (67)
14	Ho Chi Minh City, and Hanoi, Vietnam	1998–1999	? All patients	New	301/563 (53)
15	Bangkok, Thailand	1999–2000	One hospital ? selection method	? Both	90/204 (44)
8	Jakarta, Indonesia	1998–1999	Consecutive patients one clinic	? Both	31/92 (34)
Africa					
16	Senegal	1994–1995	? selection method (all Beijing were relapses)	Both	8/69 (12)
Middle East					
17	Fars Province and Tehran, Iran	1995–1996	All from Shiraz; ? random for others	Both	10/97 (10)
Europe					
11	Northwest region, Russia	1997–1998	? selection method	Both	22/100 (22)
10	Azerbaijan	1995–1996	Prison ? selection method	Both	46/65 (71)
18	Estonia	1994	Two hospitals, pulmonary TB	New	61/209 (29)
4	Netherlands	1993–1994	Whole population	Both	82/2594 (3)
19	Gran Canaria, Spain	1991–1992	Whole island	? Both	0/85 (0)
		1993			10/179 (5.5)
		1994			12/148 (8.1)
		1995			18/110 (16)
		1996			35/129 (27)
		1999			9/40 (23)
USA					
9	New Jersey	1996–1998	Whole population	Both	68/1,207 (6)
20	Houston, Texas	1994–1999	Whole population	? Both	326/1,283 (25)
Caribbean					
21	Cuba, outside Havana	1994–1995	Whole population	? Both	20/157 (13)
22	Guadeloupe	1994–1996	Whole island	? Both	1/95 (1)
22	Martinique	1995–1996	Whole island	? Both	0/31 0
South America					
22	French Guiana	1995–1996	Whole country	? Both	0/76 0

^a^N/N, number with Beijing strain/ total number of patients; ?, not clear from report.

**Table 2:** Prevalence of Beijing and W-like strains in studies not based on spoligotyping^a^

Reference	Setting	Yrs	Population	New TB or new + old	Typing methods and definitions used	Prevalence of Beijing strain N/N (%)
Asia						
23	Henan Province, China	?	No information given	?	RFLP +3.6kb *Pvu* II fragment	59/64 (92)
23	Philippines	?	No information given	?	RFLP +3.6kb *Pvu* II fragment	34/34 (100)
23	Hanoi, Vietnam	?	No information given	?	RFLP +3.6kb *Pvu* II fragment	20/50 (40)
23	Korea	1995	No information given	?	RFLP +3.6kb *Pvu* II fragment	99/138 (72)
23	Thailand	?	No information given	?	RFLP +3.6kb *Pvu* II fragment	31/49 (63)
24	Bangkok Nonthaburi, Thailand	1994–1995	Patients from 3 hospitals ? how selected. Half extrapulmonary	? Both	RFLP + comparison with Dutch database	80/211 (37)
23	Malaysia	?	No information given	?	RFLP +3.6kb *Pvu* II fragment	17/48 (35)
25	Malaysia	1993–1994	Random 3% sample from whole population	? Both	RFLP “similar” to Beijing family	83/439 (19)
Africa						
26	Cape Town, South Africa	1993–1997	Whole population	Both	RFLP “strain U”, (W-like) Two closely related patterns only	17/650 (2.6)
USA						
27	New York City	1992–1994	Patients from 5 hospitals	? Both	RFLP, strain W only	6/302 (2.0)
3	New York City	1990–1995	? selection method	? Both	RFLP, “W-like”	273/1,953 (14)
28	Central Los Angeles	1994–1996	Consecutive patients	? Both	RFLP, strain 210 (W-related)	43/162 (27)
29	California	1992–1995	All cases from specific locations	? Both	RFLP, strain 210 (W-related)	39/522 (7) 16/546 (3) 2/256 (0.8)
	Texas	1993–1995				
	Colorado	1989–1994				
2	United States (excluding NY) and Puerto Rico	1992–1997	All notified cases	Both	RFLP and/or PCR probe. Multidrug resistant W only	23/104,549 (0.02)
South America						
30	Buenaventura, Colombia	1997–1998	34 treatment failure + 73 new ? selection method	Both	RFLP + PCR probe. “Similar” to W	11/107 (10) (? 8 in new)

^a^N/N, number with Beijing strain/total number of patient; ?, not clear from report; the different typing methods are described in the introduction. RFLP restriction fragment length polymorphism (RFLP) using IS*6110.* Polymerase chain reaction (PCR) probe is a multiplex PCR probe targeted at specific insertions. The 3.6 kb *pvu*II fragment was identified by IS*1081* fingerprinting.

**Figure:**
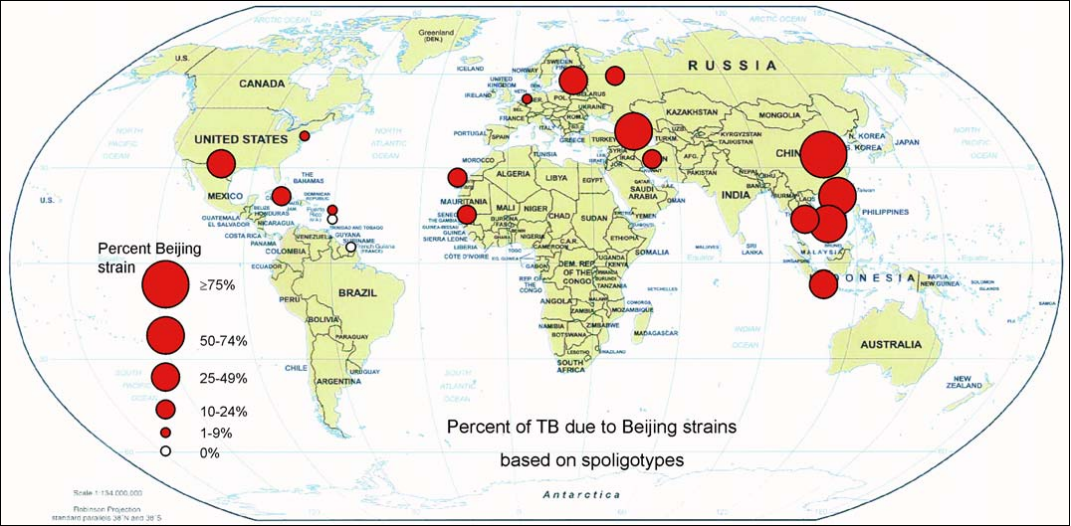
Percentage of tuberculosis due to Beijing strains. Data from studies based on spoligotyping (Table 1).

The Beijing strain was most common in the Beijing area of China, accounting for 92% of strains (4,12). The strain was common in all the Asian studies (4,8,12–15,23–25) and also in Houston, Texas (25%), and Estonia (29%) (18,20). Some examples of the Beijing family were seen in almost all the populations studied (Tables 1 and 2).

Two studies looked at trends over time (Table 1). In China, the proportion of TB due to Beijing family strains in stored specimens going back to the 1950s was similar to the proportion among more recent specimens (12). In Gran Canaria, a dramatic increase was seen from 1992 to 1996, traced to an outbreak originating from a noncompliant patient with laryngeal TB (19). In studies over a short period, variations with age can be studied as a proxy for time trends. In Vietnam, among new cases of TB, the proportion due to Beijing strains was 71% in those <25 years of age, decreasing to 41% in those >55 years (p < 0.001, chi square test for trend) (14). In Bangkok, little difference was seen with age in two studies (15,24). In Hong Kong (13), Jakarta, Indonesia (8), and Estonia (18), there was no association between age and disease due to the Beijing strain. In New Jersey, among those with tuberculosis due to W-like strains, 70% of patients were <50 years old, compared with 63% of those with other strains (p*=*0.2) (9). In Gran Canaria, the median age of cases with the Beijing strain was similar to that of all cases (19). No other studies have presented results by age.

Several studies reported associations with drug resistance (Table 3). Some studies found high rates of drug resistance among Beijing strains, but others found no difference in drug resistance profiles between Beijing and the other local strains.

**Table 3:** Association between Beijing family strains of *Mycobacterium tuberculosis* and drug resistance^a^

Reference	Place, yr	Strain		% Drug resistance									Comparison of Beijing vs. non-Beijing by drug^b^ RR 95% CI^b^
		Beijing	Non-Beijing	Any		I		S			MDR		
				Beijing	Non-Beijing	Beijing	Non-Beijing	Beijing	Non-Beijing		Beijing	Non-Beijing	
13	Hong Kong, 1998–1999	310	181			6	12	10		13			I 0.54 (0.30 to 0.97)
													S 0.76 (0.46 to 1.3)
14	Ho Chi Minh City, 1998–1999	264	235			28	19	42		19	3	2	I 1.5 (1.1 to 2.0)
													S 2.2 (1.6 to 3.0)
													MDR 1.4 (0.47 to 4.3)
15	Bangkok, 1999–2000	90	114										No assoc
8	Jakarta, 1998–1999	27	56	41	25	37	20	15		5			Any 1.6 (0.86 to 3.1)
													I 1.9 (0.92 to 3.9)
													S 2.8 (0.67 to 11.5)
16	Senegal, 1994–1995	8	61										No assoc
11	NW Russia, 1997–1998	22	78								77	58	MDR 1.3 (1.0 to 1.8)
10	Azerbaijan, 1995–1996	46	19	89	68	80	68	83		58	61	32	Any 1.3 (0.94 to 1.8)
													I 1.2 (0.84 to 1.6)
													S 1.4 (0.95 to 2.1)
													MDR 1.9 (0.96 to 3.9)
18	Estonia, 1994	61	148	70	14						34	2	Any 5.0 (3.2 to 7.6)
													MDR 17.0 (5.3 to 54.9)
19	Gran Canaria, 1991–1996	75	576	0	?								
3	New York, 1990–1995	273 (W-like)	1,680 (not W like)								93^b^	?0	p <0.001
21 ^d^	Cuba, 1994–1995	20	137	55–65	4–5	55–60	4	0–10		0.7–2	0	0.7	Any 10.8 (4.7 to 24.5)
													I 15.1 (5.8 to 38.9)
30	Colombia, 1997–1998	11	70								27	23	MDR 1.2 (0.41 to 3.4)

^a^I, isoniazid; S, streptomycin; MDR, multidrug resistant (at least isoniazid and rifampicin); blank spaces indicate that data are not available. ^b^Relative risks (RR) were calculated when possible from the data presented. These are shown with 95% confidence intervals. ^c^ Resistant to at least four drugs. Includes 206 W strains and 40 W1 strains. Identified by RFLP, not spoligotyping. ^d^Exact numbers not clear since drug resistance data only given by strain number for IS6110 defined clusters, and two Beijing strains were not clustered. For the relative risk calculation, the minimum proportion resistant among the Beijing strains was used.

An association between the successful spread of Beijing strains and BCG vaccination has been suggested (4). In Jakarta, Indonesia (8), 26% of those with Beijing strains and 23% of other patients had a BCG scar. In Vietnam, although a higher proportion of those with Beijing strains than with other strains had a BCG scar, this association was no longer apparent after the data were adjusted for age (14).

## Discussion

This review has confirmed the ubiquity of the Beijing family of strains. Only a few of the smaller studies (in Martinique and French Guiana) found no examples, and the proportion of TB due to Beijing strains in several Asian studies was >50%. However, studies could only be included in the review if they mentioned the Beijing strain or strain W or presented data showing spoligotypes. Some of the excluded studies may have found Beijing strains but not reported them as such (33,34). Others may have looked for Beijing strains but not reported negative findings. The only articles identified that reported not finding Beijing strains were studies including more than one study site. It is not known how unusual it is for a genotype family of *M. tuberculosis* to be as widespread as this. Comparable data are not available for other strains, although they are beginning to be gathered, and some other strains have also been found in several distinct settings (35).

In many studies, the true proportion of TB attributable to the Beijing family of strains is hard to assess. Difficulties arise due to the variable strain definitions used and the way patients were selected for inclusion. Spoligotyping seems to be both sensitive and specific for the Beijing family and is also easily compared between studies (6). Although IS*6110* fingerprinting can also be used to detect this genotype family, with results that correlate closely with the spoligotypes, most published studies have used narrow definitions, based on a single strain or a few closely related strains defined by IS*6110* fingerprinting; such studies are thus likely to underestimate the prevalence of Beijing strains. Studies including drug resistance in the definition (2) and those that appear to have defined the strains after grouping by drug resistance (26) may also underestimate the prevalence.

Some of the studies (those in the Netherlands, New Jersey, Houston, Texas, and French Guiana and the Caribbean islands) included information on all TB patients in the population and thus provide reliable estimates of prevalence. Others were less representative, and many did not state how the patients were selected (Table 1 and 2). Studies that included patients from particular hospitals may be representative of an area, but referral hospitals may be biased if they accept a high proportion of drug-resistant or complex cases. Similarly, convenience samples may not be representative of the community of TB patients, particularly if the samples were kept because they were interesting in some way (e.g., drug resistant or from epidemiologically related cases). TB patients in prison (10) may not have the same strains as those in the community. Some studies included only new patients, and others included both new patients and recurrent cases. This distinction, which was often not clear in the reports, could influence the results if relapse rates differ between strains.

In many studies, some culture-positive specimens are not typed because they are nonviable. IS*6110* RFLP typing relies on large quantities of DNA and hence on viable strains, and theoretically some genotypes may survive better than others in vitro. Spoligotyping is PCR-based so does not require viable isolates, but it is sometimes used only as a secondary method in specimens that have already been typed by IS*6110* RFLP.

Associations with drug resistance were variable (Table 3): of the 12 studies with data available, only 4 found statistically significant increases in the proportions of drug resistance among those with Beijing strains. Of the Asian studies, only one found a statistically significant increase in drug resistance in Beijing strains (14), and in Hong Kong the Beijing strains were less likely than the others to be isoniazid resistant (13). In contrast, Beijing strains were strongly associated with drug resistance in New York, Cuba, and Estonia (3,18,21). In New York, the spread of the W strain, which was mainly nosocomial and institutional, has been attributed in part to drug resistance. Once a strain has become multidrug resistant, treatment is more complicated so patients may remain infectious for a longer period. Whether the Beijing family has a particularly high probability of acquiring drug resistance is not known but is suggested by the fact that these associations with the same strain family have been found in widely distributed areas.

The published studies provided little direct evidence that the Beijing strain has been increasing. Of the two studies that included time trends, one found no increase in a population with a very high prevalence for many decades (12), and in the other the increase may be attributable to the characteristics of the index patient in the outbreak (19,36). In Vietnam, the proportion of new TB patients with the Beijing strain decreased with age, suggesting an increase in Beijing strains in the communities studied (14). No association with age was found anywhere else (8,9,13,15,18,19,24), including the two other studies restricted to new patients (13,18).

On the other hand, the ubiquity of the Beijing strain and its frequent appearance in outbreaks, particularly of drug-resistant TB, suggest that it may have the potential to spread. In Estonia, although there was no association between Beijing strains and age, TB and particularly multidrug-resistant (MDR) TB have been increasing, and most MDR TB was found to be due to Beijing strains (18). The limited amount of information available from most areas of the world and the possible biases in many of the studies make definite conclusions about the extent of spread and associations with drug resistance impossible. Through the European Concerted Action on New Generation Genetic Markers and Techniques for the Epidemiology and Control of Tuberculosis, a standard definition of the Beijing genotype is being finalized, by comparisons of large collections of strains typed with spoligotyping, IS*6110* RFLP, and Region A RFLP, which visualizes insertion of IS*6110* in the genomic dnaA-dnaN locus (ms. in preparation). Studies are planned to reanalyze available data worldwide by using standard definitions and approaches.

Further studies are also needed to include more areas in an unbiased way, to study historical specimens if possible, and to investigate the virulence (8) and transmissibility of this potentially important family of *M. tuberculosis* strains. The question to be answered is if and to what extent Beijing genotype strains have selective advantages over other *M. tuberculosis* genotypes in the ability to gain resistance and to interact with the host immune defense system. If Beijing genotype strains represent a higher level of evolutionary development of *M. tuberculosis* being selected for as a result of the introduction of tuberculostatics, which inhibit the growth of *M. tuberculosis*, then consequences for the treatment of tuberculosis will be serious.
